# Ultrastructural and immunocytochemical evidence of a colonial nervous system in hydroids

**DOI:** 10.3389/fncir.2023.1235915

**Published:** 2023-09-07

**Authors:** Igor A. Kosevich

**Affiliations:** Department of Invertebrate Zoology, Faculty of Biology, Lomonosov Moscow State University, Moscow, Russia

**Keywords:** Cnidaria, Hydrozoa, colonial nervous system, coenosarc, *Clava multicornis*, *Dynamena pumila*, *Obelia longissima*

## Abstract

**Background:**

As the sister group to all Bilateria, representatives of the phylum Cnidaria (sea anemones, corals, jellyfishes, and hydroids) possess a recognizable and well-developed nervous system and have attracted considerable attention over the years from neurobiologists and evo-devo researchers. Despite a long history of nervous system investigation in Cnidaria, most studies have been performed on unitary organisms. However, the majority of cnidarians are colonial (modular) organisms with unique and specific features of development and function. Nevertheless, data on the nervous system in colonial cnidarians are scarce. Within hydrozoans (Hydrozoa and Cnidaria), a structurally "simple" nervous system has been described for *Hydra* and zooids of several colonial species. A more complex organization of the nervous system, closely related to the animals' motile mode of life, has been shown for the medusa stage and a few siphonophores. Direct evidence of a colonial nervous system interconnecting zooids of a hydrozoan colony has been obtained only for two species, while it has been stated that in other studied species, the coenosarc lacks nerves.

**Methods:**

In the present study, the presence of a nervous system in the coenosarc of three species of colonial hydroids - the athecate *Clava multicornis*, and thecate *Dynamena pumila* and *Obelia longissima* - was studied based on immunocytochemical and ultrastructural investigations.

**Results:**

Confocal scanning laser microscopy revealed a loose system composed of delicate, mostly bipolar, neurons visualized using a combination of anti-tyrosinated and anti-acetylated a-tubulin antibodies, as well as anti-RF-amide antibodies. Only ganglion nerve cells were observed. The neurites were found in the growing stolon tips close to the tip apex. Ultrastructural data confirmed the presence of neurons in the coenosarc epidermis of all the studied species. In the coenosarc, the neurons and their processes were found to settle on the mesoglea, and the muscle processes were found to overlay the nerve cells. Some of the neurites were found to run within the mesoglea.

**Discussion:**

Based on the findings, the possible role of the colonial nervous system in sessile hydroids is discussed.

## Introduction

As a sister group to all Bilateria, representatives of the phylum Cnidaria, such as sea anemones, corals, jellyfishes, and hydroids, possess a recognizable and well-developed nervous system and have attracted considerable attention over the years from neurobiologists and evo-devo researchers (Anderson, 2004). The application of multiple electrophysiological and other methods has provided us with a great deal of information about the scope and composition of the cnidarian nervous system.

For a long time, the prevailing opinion has been that the layout of the cnidarian nervous system is that of a diffuse, two-dimensional network of cells combining sensory and motor functions whose processes are not differentiated into axons and dendrites, and which conduct impulses in any direction (Mackie, 1990). Investigation of the representatives of different cnidarian taxa has led to the description of the nerve cells found in both the epidermis and the endoderm, although they are much more abundant in the epidermis (Grimmelikhuijzen and Westfall, 1995; Kass-Simon and Hufnagel, 2015; Kelava et al., 2015; Rentzsch et al., 2019).

Within hydrozoans (Hydrozoa, Cnidaria), a structurally “simple” nervous system was described for *Hydra* (e.g., Burnett and Diehl, 1964; Lentz and Barrnett, 1965; Davis et al., 1968) and the zooids of several colonial (modular) species (e.g., Jha and Mackie, 1967; Stokes, 1974a; Kosevich, 2013; Mayorova and Kosevich, 2013). A more complex organization of the nervous system closely related to the animals' motile mode of life was shown for the medusa stage (e.g., Jha and Mackie, 1967; Singla, 1978a,b; Mackie and Meech, 1995; Lin et al., 2001; Koizumi et al., 2015). The zooids of several colonial hydrozoans (e.g., Josephson, 1961b, 1965; Spencer, 1974; Stokes, 1974b) and medusae (e.g., Spencer, 1974; Mackie and Meech, 1985, 1995; Mackie, 2004) were used as model objects for electrophysiological studies of nervous system activity in this group of invertebrates.

Immunocytochemical and molecular studies have revealed that on the whole, the “simple” nervous system of hydroids and cnidarians is not so simple and is constructed based on distinct nerve cells that utilize different neurotransmitters (e.g., Grimmelikhuijzen et al., 1991; Grimmelikhuijzen and Westfall, 1995; Koizumi, 2002, 2016) and diverse types of sensory cells (e.g., Westfall and Kinnamon, 1978; Singla, 1983). The basiepidermal nerve net comprises interneurons with their perikarya and neurites, as well as neurites that extend inwards from epidermal sensory cells. Even from the morphological viewpoint, the nerve cells of cnidarians can be classified into unipolar, bipolar, tripolar, or quadripolar (multipolar) neurons and sensory cells (Westfall and Epp, 1985; Havrilak et al., 2017). It became clear that the cnidarian polyp (zooid) typically has several partly overlapping molecularly defined subsets of the nervous system, differing in terms of the neuropeptides used, function, etc. (Mackie, 1990; Koizumi, 2002; Koizumi et al., 2004; Sprecher, 2022).

**Figure 1:**
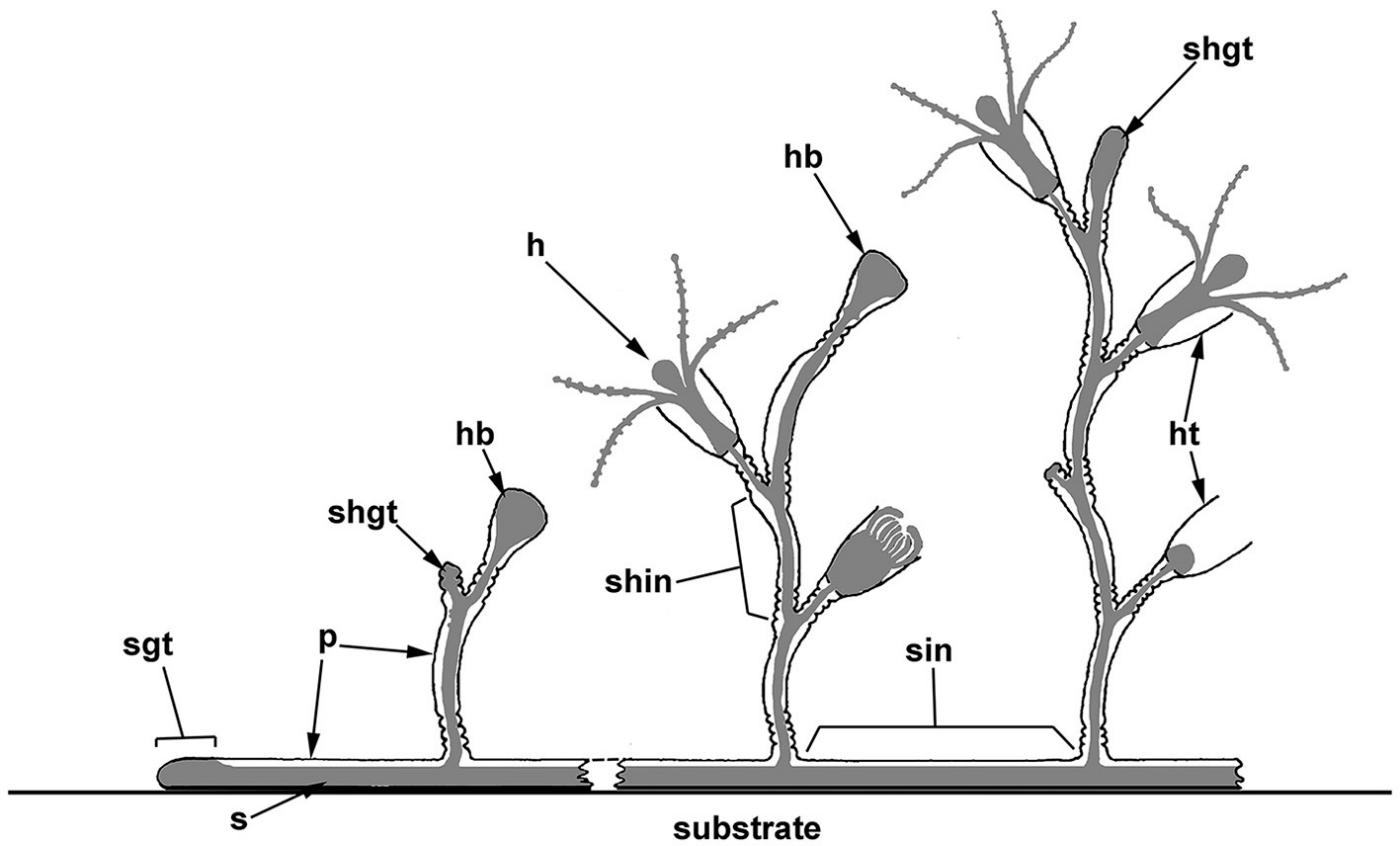
Scheme of the polypoid colony organization in thecate hydroids, and the spatial relationship between the soft tissues and the outer skeleton (perisarc). The colony consists of tubular stolons that grow over the substrate, as well as shoots branching from the stolons, which carry numerous zooids (hydranths). Coenosarc (a tubular tissue composed of two epithelial layers) and hydranths are shown in gray. h, hydranth; hb, hydranth bud (rudiment); ht, hydrotheca; p, perisarc; s, stolon; shgt, shoot growing tip; shin, shoot internode; sgt, stolon growing tip; and sin, stolon internode.

**Figure 2:**
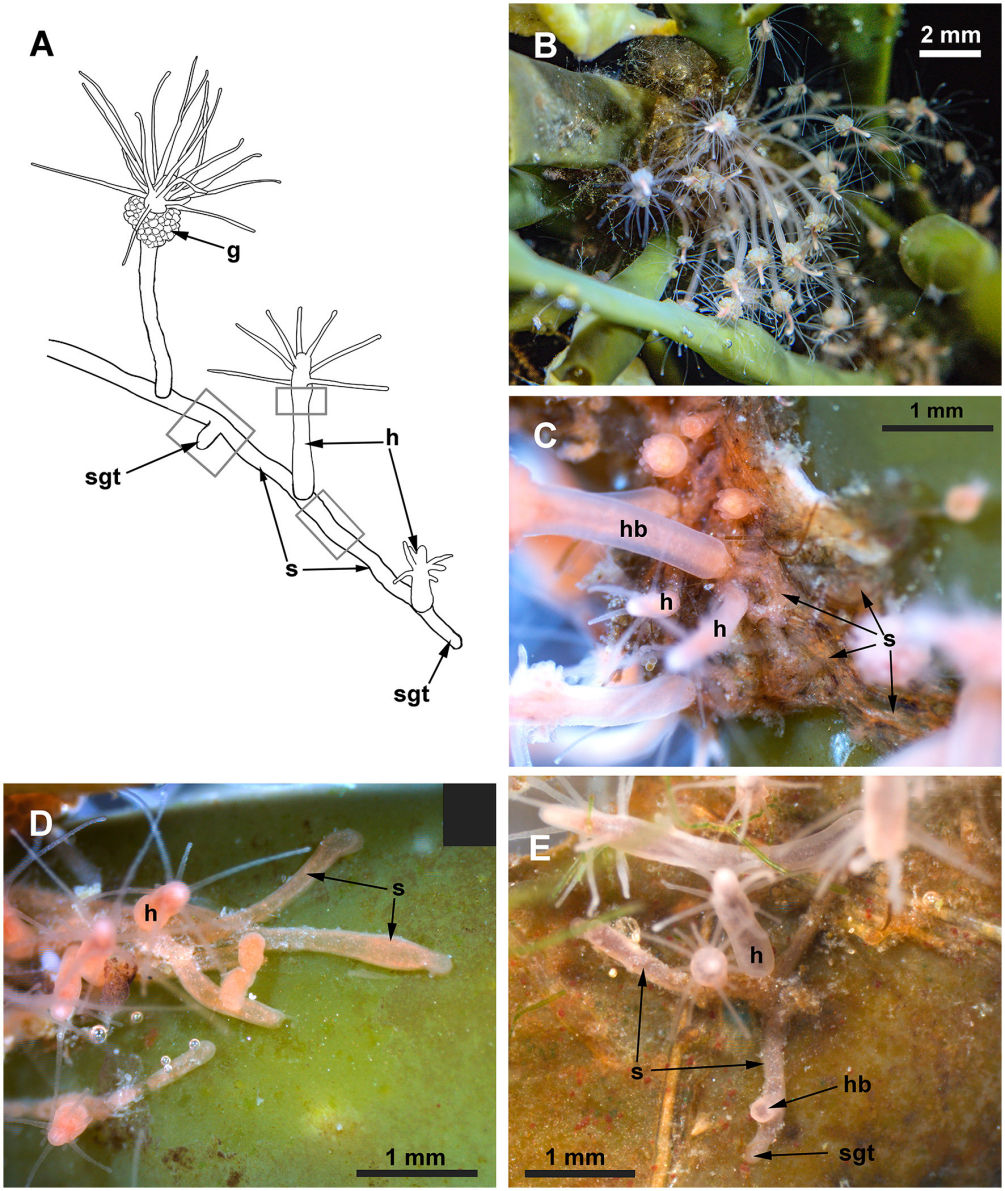
*Clava multicornis* colony. **(A)** Scheme of a colony growing on a flat and free substrate. **(B)** The small colony growing on the brown algae *Ascophyllum nodosum*. The compact colony occupies the node of the frond. **(C)** Close view of the interlacing stolons covered with the perisarc, while the hydranths are “naked.” **(D, E)** Distal parts of stolons growing straight at the periphery of the colony over the free substrate. g, gonads; h, hydranth; hb, hydranth base; s, stolon; and sgt, stolon growing tip. Gray rectangles show the colony parts used for the investigation.

**Figure 3:**
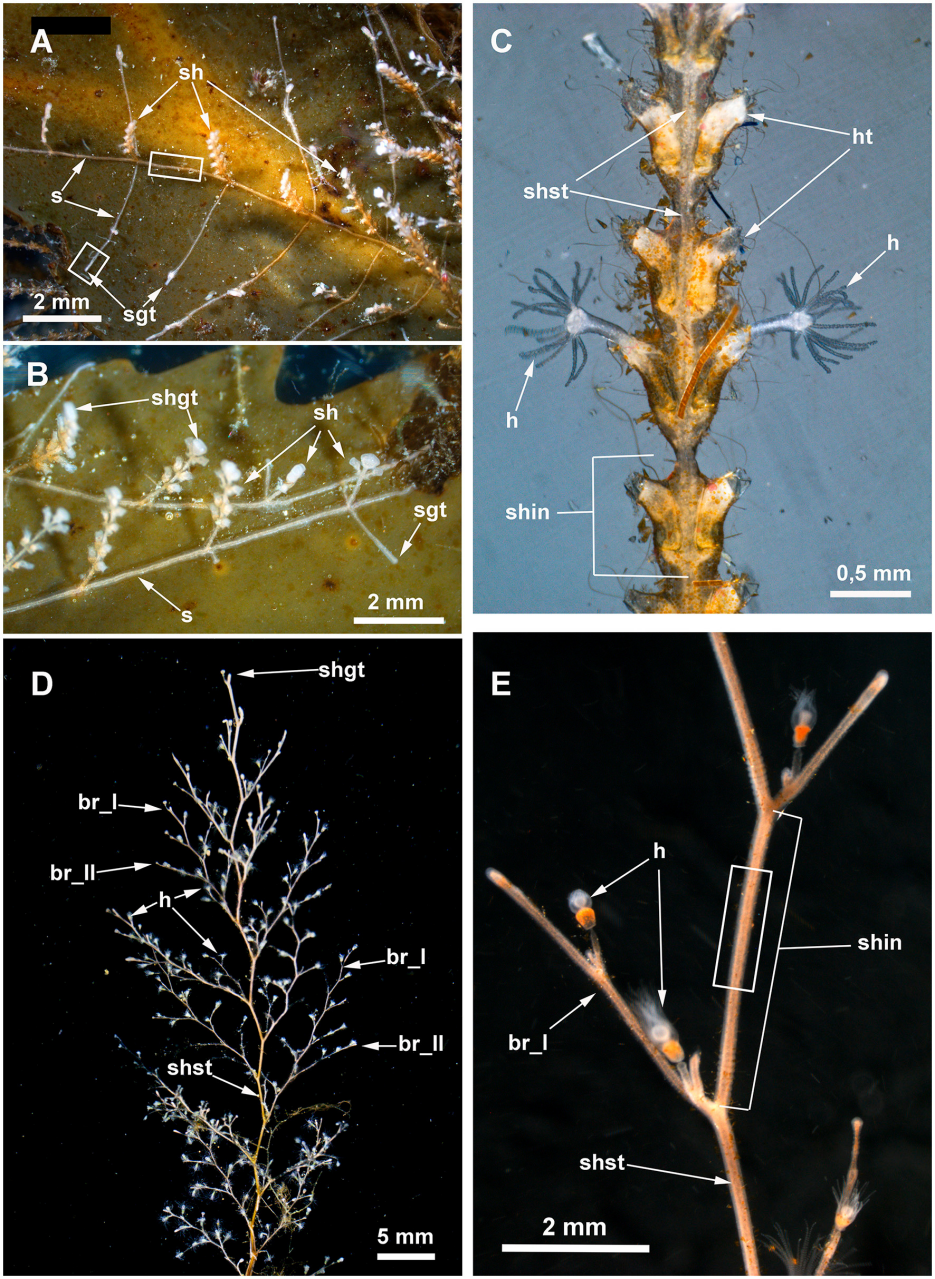
Details of *Dynamena pumila* **(A–C)** and *Obelia longissima* **(D, E)** colonies. White rectangles indicate the colony elements used for the study. **(A)** General view of the peripheral part of the *D. pumila* colony showing the straight-line mode of stolon growth and the regular branching of stolons from the bases of the shoots. **(B)** Close view of the peripheral part of the colony with young shoots with a monopodial mode of growth. **(C)** Part of *D. pumila* shoot illustrating its organization: the two opposite rows of hydrothecae partly fused with the shoot stem; the shoot is subdivided into equal internodes, each composed of a pair of hydrothecae. **(D)** General view of the characteristic large shoot of *O. longissima* with regular branching. **(E)** Close view of a part of a *O. longissima* shoot depicting the large straight internodes of the stem and the smaller internodes of lateral branches. br_I, the first order branch; br_II, branch of the second order; h, hydranth; ht, hydrotheca; sh, shoot; shgt, shoot growing tip; shin, shoot internode; shst, shoot stem; sgt, stolon growing tip.

The colonial polypoid stage of sessile hydrozoans (Hydrozoa, excluding Siphonophorae) is mostly presented by tubular stolons creeping over the substrate and single hydranths (feeding zooids) or shoots carrying several or many hydranths that branch from the stolons and protrude into the surrounding water (Figures 1, 2A, 3A, B, D). Zooids or shoots settle on the stolons at certain intervals (Marfenin and Kosevich, 2004; Bouillon et al., 2006). The hydranths of the colony are interconnected by the coenosarc—the tubular colonial tissue responsible for the distribution of food in colony parts (Figure 1) (Marfenin, 2015; Marfenin and Dementyev, 2017). The coenosarc of the stolons, zooid pedicels, and internodes of the shoots in all colonial hydroids is covered by a rigid outer skeleton—the chitinous perisarc—that determines most of the characteristic morphological features of colonial hydroids (Figures 1, 3) (Naumov, 1969; Bouillon et al., 2006).

Despite a long history of cnidarian nervous system investigation, data on the nervous system in colonial forms are scarce. The presence of the colonial nervous system was shown for some octocorals (e.g., Dickinson, 1978; Satterlie et al., 1980; Pernet et al., 2004) and siphonophores (e.g., Jha and Mackie, 1967; Mackie, 1973; Grimmelikhuijzen et al., 1986; Norekian and Meech, 2020), while only indirect data, often based on electrophysiological studies, provide evidence of its presence in colonial hydroids (Josephson, 1961a,b, 1962, 1965). Furthermore, electrophysiological studies have demonstrated the ability of certain tissues in hydrozoans (and cnidarians as a whole) to conduct electrical stimuli without the presence of nerve fibers. It was suggested that this happened via cells of the epithelial layer or the muscular processes of epithelial cells (Josephson, 1965). However, for a long time, there were problems with histological evidence on muscular tissue in hydroid stolons (Josephson, 1961b).

Direct evidence of a colonial nervous system (nervous system in the coenosarc of the colony) interconnecting zooids of a hydrozoan colony was obtained only for athecate *Cordylophora* and *Hydractinia* (Bullock and Horridge, 1965; Stokes, 1974a), while it was stated that in other species (e.g., *Tubularia, Syncoryne*, and *Proboscidactyla flavicirrata*), stolons lack nerves (Bullock and Horridge, 1965; Spencer, 1974). One possible explanation is the presence of the chitinous perisarc that prevents an immunocytochemical investigation of the nervous system, as it is impermeable to antibodies. Simultaneously, it is astonishing that the nerve elements were never reported in ultrastructural investigations of the hydrozoans' coenosarc.

In the present study, the presence of a nervous system in the coenosarc of three species of colonial hydroids was confirmed based on immunocytochemical (ICC) and ultrastructural investigations. To apply ICC methods, the perisarc of stolons of athecate *Clava multicornis* (Forsskål, 1775) and thecate *Dynamena pumila* (Linnaeus, 1758), as well as of the shoot internodes of thecate *Obelia longissima* (Pallas, 1766), was first perforated. Confocal laser microscopy revealed a sparse (loose) system composed of delicate, mostly bipolar neurons with neurites predominantly running longitudinally. Multipolar neurons were rare. Only ganglion nerve cells were observed. Intriguingly, the neurites were observed in the growing stolon tips close to the tip apex. Ultrastructural data confirmed the presence of neurons in the coenosarc epidermis of all the studied species. The microtubules and dense-core vesicles characterized the neurons of the coenosarc in all the species. The location of the soma and neurites differed from that characteristic of zooids or solitary polyps. Within the latter, the neurons and their neurites lay on a sheet of muscle processes. In the coenosarc, the neurons and their processes settled on the mesoglea, and the muscle processes overlay the nerve cells. Some of the neurites could run within the mesoglea.

## Materials and methods

### Animals

In the present study, the coenosarc of three widespread hydroid species was studied. The choice of the objects was determined based on the accessibility of the samples, the presentation of different colony types (stolonial vs. shoot-forming), and the different modes of shoot growth (sympodial-monopodial). One species with thinner and softer perisarc belongs to the order Anthoathecata (athecate hydroid with a stolonial colony), while two other species are representatives of the order Leptothecata (thecate hydroids with shoot-forming colonies and a different mode of shoot growth) and have a well-developed and rigid perisarc (Marfenin and Kosevich, 2004; Kosevich, 2012, 2015).

*Clava multicornis* (Forsskål, 1775) (Hydractiniidae, Anthoathecata) is a widespread species inhabiting the intertidal zone; it is found mostly on brown algae in the temporal waters of the North Atlantic Ocean, the Mediterranean and Adriatic seas, and some Arctic seas. It is considered an Atlantic broad boreal-arctic species (Antsulevich, 2015). The hydroid is characterized by stolonial colonies with relatively large hydranths settled on the stolons.

*Dynamena pumila* (Linnaeus, 1758) (Sertulariidae, Leptothecata) is a high boreal Atlantic species, typically found in the lower intertidal zone and sometimes deeper on a variety of plant and inorganic substrates (Antsulevich, 2015). It forms colonies with branching shoots characterized by monopodial growth.

*Obelia longissima* (Pallas, 1766) (Campanulariidae, Leptothecata) is a high boreal and arctic species, recorded from the upper sublittoral zone on a variety of animal, plant, and inorganic substrates (Antsulevich, 2015). The species typically forms colonies with large branched shoots characterized by a sympodial mode of growth and a thick perisarc of the shoot internodes (Kosevich, 1991b).

All cnidarians possess a unique type of cell—the stringing cells or nematocytes. In colonial hydroids, the differentiation of nematocytes begins predominantly in the coenosarc of stolons (Kossevitch, 1999; Kosevich, 2013). At the beginning of cyst differentiation, they are non-functional and cannot fire until final maturation. Nematocytes with non-functional cysts are called nematoblasts. The not fully developed cyst is located in the middle of the nematoblast. Nematoblasts are found at the base of the epidermis migrating to the final location. At the final location (e.g., tentacles of the hydranths), they nest themselves in the epidermis. Their cysts mature, and the cells on the whole acquire the state of nematocytes. Only a fully developed cyst at the final location of the nematocyte can fire.

Samples of all species on natural substrates were collected in the intertidal and upper sublittoral zones in the vicinity of the N.A. Pertsov White Sea Biological Station (Lomonosov Moscow State University) (66° 34′ N, 33° 08′ E). In the laboratory, the colonies were kept in aquariums with natural seawater under 12°C and fed daily with newly hatched *Artemia salina (Linnaeus, 1758)* nauplii.

### Immunocytochemistry

For the ICC investigation of stolons, colonies were grown on cover glasses from a single shoot or hydranth. When the new colonies formed stolons and 1–2 new hydranths/shoots, they were anesthetized with an isotonic solution of MgCl_2_ for 5 min and fixed with 4% paraformaldehyde (PFA; Fluka, Germany) in phosphate-buffered saline (PBS; Fluka, Germany) at 4°C for 24 h directly on the cover glasses. All the following procedures were applied to the entire sample on the cover glasses.

For ICC investigation of the coenosarc of the shoot parts including 2-3 internodes were dissected from the entire colony, anesthetized with an isotonic solution of MgCl_2_ for 5 min, and fixed with 4% paraformaldehyde (PFA; Fluka, Germany) in phosphate-buffered saline (PBS; Fluka, Germany) at 4°C for 24 h.

The fixed material was washed three times for 1 h each in PBS. Before immunostaining, parts of the colony enclosed by the perisarc (stolons and shoot internodes) needed partial dissection of the perisarc with a scalpel to allow antibodies to penetrate into the tissues. Sometimes, this caused local damage to the tissues.

After a brief wash with PBS, the samples were incubated in a block solution (**BS**) containing 1% bovine serum albumin (BSA; Sigma, St. Louis, MO, USA), 0.1% cold water fish skin gelatin (Sigma), 0.5% Triton-X100 (Ferak Berlin, Germany), and 0.05% Tween 20 (Sigma) for 24 h. Subsequently, they were incubated at +4°C for 48 h in a mixture of primary antibodies, which included mouse monoclonal anti-tyrosinated α-tubulin (1:1,000; Sigma Cat # T9028) and mouse monoclonal anti-acetylated α-tubulin (1:2,000; Sigma Cat #T6793) antibodies), with or without the addition of rabbit polyclonal anti-RF-amide antibodies (Grimmelikhuijzen et al., 1996, 2004; Plickert and Schneider, 2004) (1:500; a gift from Prof. G. Plickert, Cologne) diluted in BS.

The samples were then rinsed of primary antibodies by washing four times for 3 h each in BS. They were incubated for 48 h at 4°C in the following mixture of secondary antibodies: Donkey Anti-Mouse IgG Antibodies labeled with Alexa Fluor 555 (1:500; Molecular Probes, # A-31570) and Donkey Anti-Rabbit IgG Antibodies labeled with Alexa Fluor 647 (1:500; Molecular Probes, #A10040).

Next, the samples were washed at 4°C in BS (three times for 2 h each), rinsed with PBS, and stained for 3 h with a mixture of DAPI (100 ng/ml; Sigma) and BODIPY FL phallacidin (1:100; # B607; Molecular Probes). Following a brief (20 min) wash in PBS, the shoot samples were mounted on a cover slip coated with poly-L-lysine (Sigma-Aldrich, St. Louis, MO, USA) and were then cleared and mounted in Murray Clear (a 2:1 mixture of benzyl benzoate and benzyl alcohol) (von Dassow, 2010). The stolon samples on the cover glasses were cleared and mounted in Murray Clear. They were mounted on large (22 x 60 mm) cover glasses (Menzel-Glaser): so they were mounted between two cover glasses.

The samples were studied using a Nikon A1 confocal microscope (Tokyo, Japan) (White Sea Biological Station, Russia). Z-projections were generated using NIS-Elements D4.50.00 (Nikon) and Image J V.1.54d (https://imagej.org), and they were processed with Adobe Photoshop 2020 v. 21.2.4 × 64 (Adobe Systems, San Jose, CA, USA).

Negative controls included specimens processed without incubation in primary antibodies. They revealed bright, non-specific labeling of the damaged tissues, but they did not reveal any unspecific labeling of the undamaged tissues. Therefore, only undamaged tissues were analyzed. The autofluorescence control was prepared without adding a fluorochrome (secondary antibodies) and showed weak, non-specific fluorescence.

### Transmission electron microscopy

For electron microscopy, part of the hydroid colony along with a piece of natural substrate (brown algae) was fixed overnight at 4°C in 2.5% glutaraldehyde (Ted Pella, Inc.) in 0.1 M Na-cacodylate buffer (0,1 M Na-cacodylate, 85,55 mM NaCl, 5 mM CaCl_2_, 5 mM MgCl_2_, and pH 7.4). The fixed material was washed three times for 1 h each in the same buffer and then postfixed for 2 h in 1% OsO_4_ (Spi Supplies, West Chester) in the same buffer. After washing the specimens with the same buffer, further preparation included dehydration in ethanol series and acetone, as well as embedding in Epon-Araldite resin (Electron Microscopy Sciences, Fort Washington, PA, USA). Semi-thin sections with a thickness of 0.5 μm and thin sections with a thickness of 70–80 nm were cut using a Leica EM UC6 ultratome (Leica, Germany). The semi-thin sections were stained with a mixture of methylene blue and toluidine blue (Mironov et al., 1994) and were observed under a Leica DM 2500 stereomicroscope, which was equipped with a Leica DMC 2900 digital camera (Leica, Germany).

The ultrathin sections were stained in uranyl acetate, followed by lead citrate, and they were examined using JEM-1011 JEOL and JEM-1400 Flash JEOL transmission electron microscopes (JEOL, Akishima, Japan). For TEM, sections of stolons from at least two different colonies of each species were examined.

Digital images were processed using Adobe Photoshop 2020 v. 21.2.4 × 64 (Adobe Systems, San Jose, CA, USA).

## Results

### Clava multicornis

In *C. multicornis*, hydranths develop directly on the stolons (Figure 2). A relatively thin and non-rigid perisarc covers the entire stolonial system of the colony and the bases of the hydranths. The hydranths are naked—their epidermis is covered with only an invisible layer of glycocalyx. In nature, the stolons of the colony do not grow straight but constantly change the direction of their growth. However, in the lab, the colonies growing on artificial substrates have straighter stolons. The coenosarc of the stolons occupies mostly the entire space within the perisarc tube. That is why it was impossible to disrupt the perisarc without traumatizing the tissue. This resulted in some artifacts: non-specific labeling of the injured tissue during ICC studies.

**Figure 4:**
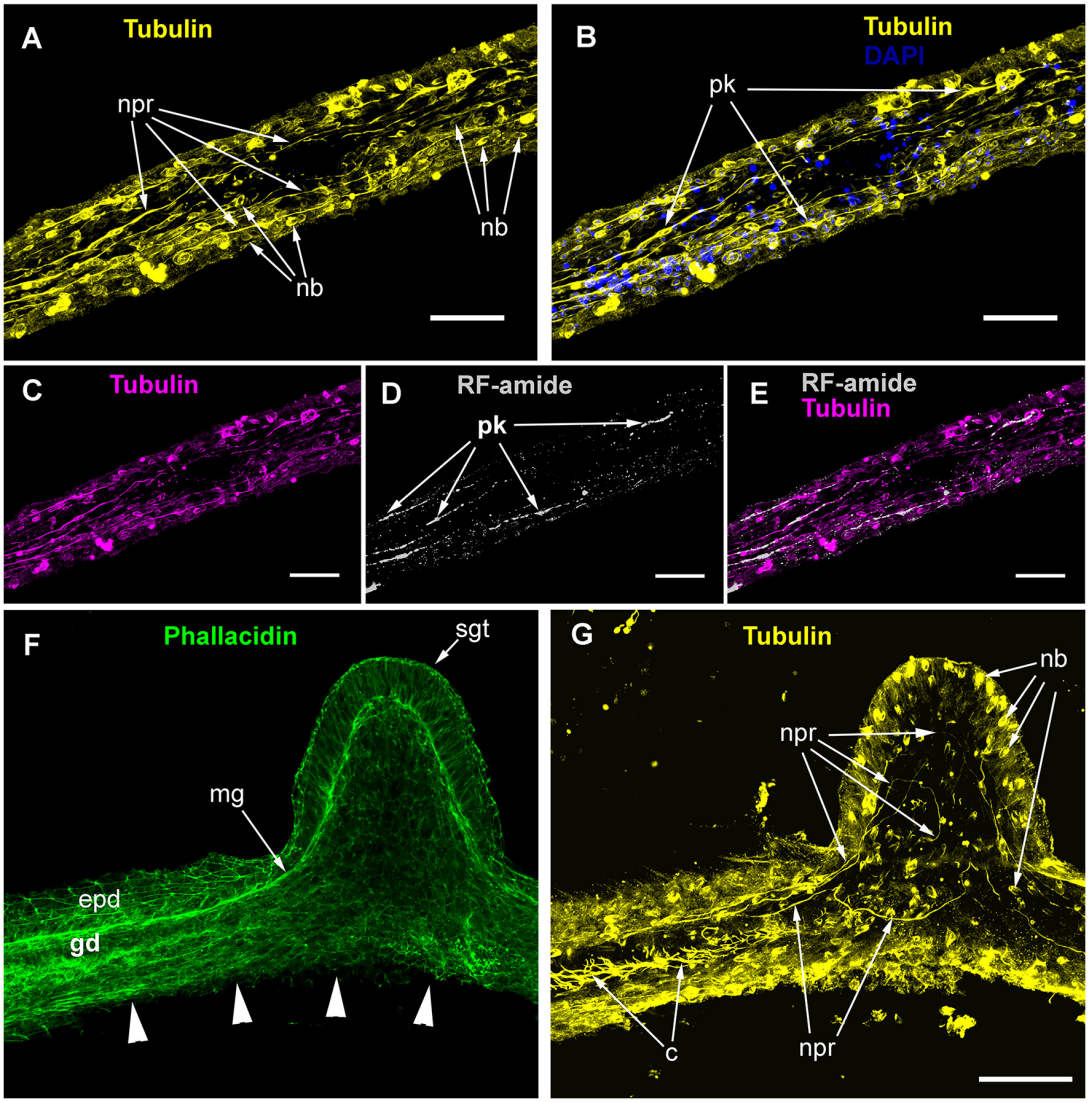
Alpha-tubulin-like and anti-RF-amide-like immunoreactivity in the stolon of *Clava multicornis*. Confocal laser scanning microscopy. Maximum Z-projections **(A–E)** after immunostaining against alpha-tubulin shown in yellow **(A, B, G)** or magenta **(C, E)**, and RF-amide shown in white **(D, E)**. Nuclear staining (DAPI) shown in blue **(B)** and phallacidin staining shown in green **(F)**. **(A, B)** A straight part of the stolon between the hydranth bases with alpha-tubulin-like immunoreactive cells. **(C–E)** The same part of the stolon (and the same stack of optical sections) showing overlapping of the alpha-tubulin-like and the anti-RF-amide-like immunoreactivity. (**F, G)** Part of a stolon with the lateral growing tip; staining with phallacidin **(F)** helps visualize the outer shape and the borders of the epithelia layers; immunostaining against alpha-tubulin **(G)** shows neuron-like cells and processes within the new growing tip, as well as numerous nematoblasts. c, cilia; epd, epidermis; gd, gastrodermis; mg, mesoglea; nb, nematoblasts; npr, nerve process; pk, perikaryon; and sgt, stolon growing tip; arrowheads point to the place where the perisarc was disrupted that caused the disturbance of the epidermis integrity. **(A–E)** Maximum Z-projections of 38 optical sections with 0.3 μm step (overall section thickness-−11.1 μm). **(F, G)** Maximum Z-projections of 56 optical sections with 0.2 μm step (overall section thickness-−11.0 μm). Scale bar – 50 μm.

The ICC labeling of the stolons with anti-α-tubulin antibodies revealed the system of scattered cells with delicate processes (Figures 4A, B). The processes predominantly run longitudinally—along the main axis of the stolon. Most of the alpha-tubulin-like immunoreactive cells resemble bipolar neurons. Multipolar neuron-like cells are rare. The system of alpha-tubulin-like immunoreactive cells forms a loose network without any signs of condensation. Besides labeling nerve-like cells, the anti-α-tubulin antibodies clearly visualized numerous nematoblasts within the stolons. Sometimes, it appears that the nematoblasts are located on or along the processes of the neuron-like cells.

Simultaneous labeling with anti-α-tubulin and anti-RF-amide antibodies revealed that most of the alpha-tubulin-like immunoreactive cells also displayed anti-RF-amide-like immunoreactivity (Figures 4C–E). The anti-RF-amide antibodies clearly marked the cell bodies (Figure 4D), while the cell processes were marked in an interrupted manner—mostly like a dotted line.

Labeling of the stolon at the base of the growing tip of a new lateral stolon revealed alpha-tubulin-like immunoreactive cells and processes that reached the base of the epidermis, practically at the apex of the tip (Figures 4F, G).

The ICC labeling of *C. multicornis* stolons showed that the length of neurite-like processes was ~50–100 μm and that they ran approximately 10 μm aside one another.

**Figure 5:**
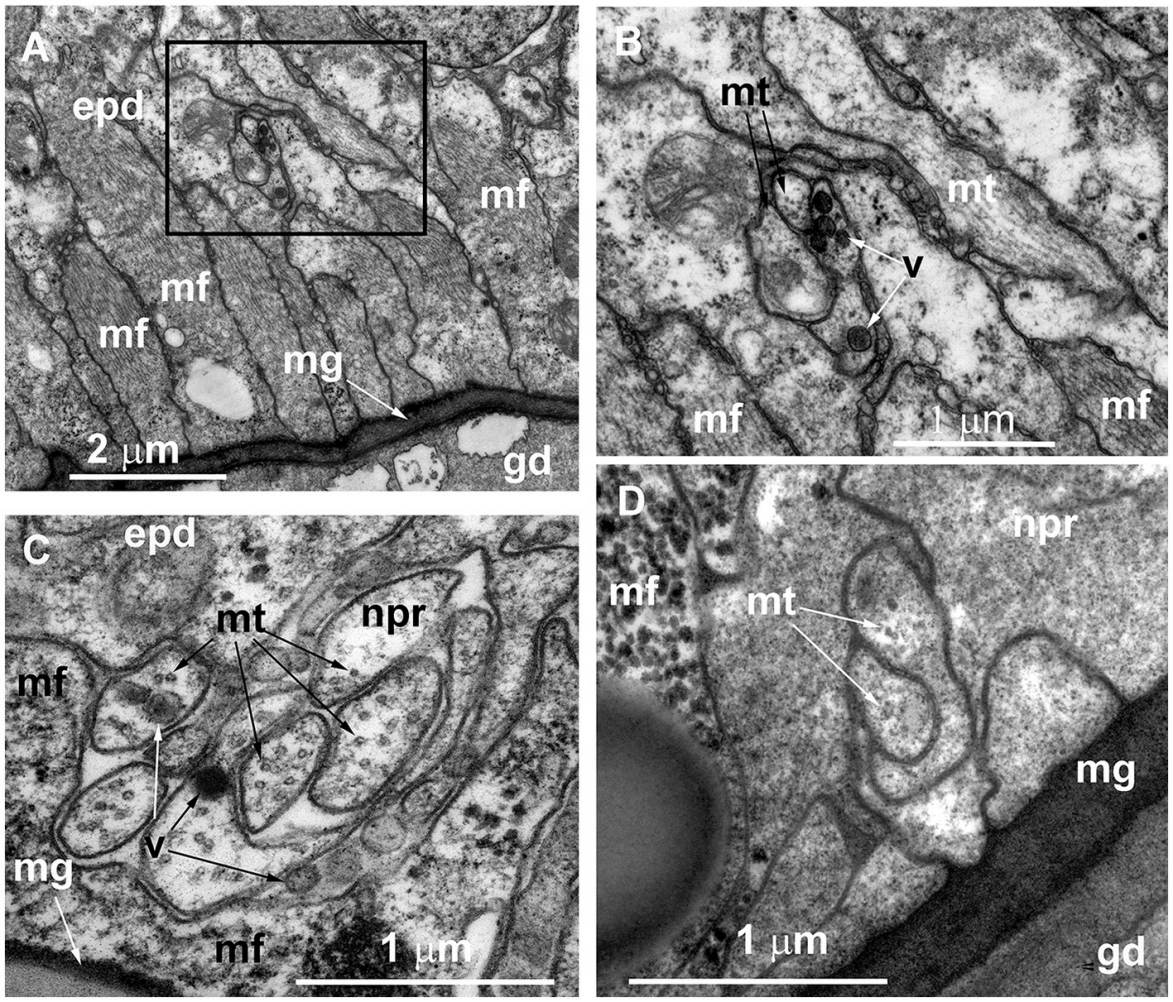
Organization of the basal part of the epidermis at the base of the hydranth **(A–C)** and in the stolon **(D)** of *Clava multicornis*, transverse ultrathin sections, TEM. **(A)** Sheet of epidermal longitudinal muscular processes over the mesoglea at the basal part of the hydranth (the section runs slightly oblique). **(B)** The enlarged part marked on A showing the neurite bundle. **(C)** The neurite bundle at the base of the hydranth epidermis separated from the mesoglea by the muscular processes. **(D)** The neurite bundle in the stolon epidermis in direct contact with the mesoglea and superimposed by muscular processes. epd, epidermis; gd, gastrodermis; mf, muscular microfilaments; mg, mesoglea; mt, microtubules; npr, nerve process; and v, vesicles.

During the ultrastructural investigation, cross-sections of stolons were compared with a cross-section of the hydranth base. In the latter, the entire layer of longitudinal muscular processes of the epitheliomuscular cells represents the basal part of the epidermis that contacts the mesoglea (Figures 5A, C). The neurite-like processes predominantly form the bundles that run above the muscular sheet (Figures 5A–C). The neurite-like processes can be distinguished by the presence of microtubules and dense-core and clear vesicles (Figures 5B, C). In contrast to the hydranth base, the neurite-like processes in the epidermis of stolons lie directly in contact with the mesoglea underneath the longitudinal muscular processes of the epitheliomuscular cells (Figure 5D). The neurite-like processes of stolons rarely form bundles and are characterized by the presence of microtubules.

### Dynamena pumila

The colonies of this species develop branching shoots that are characterized by monopodial growth. All parts of the colony are covered with rigid perisarc. The perisarc also forms hydrothecae, which serve as protective housing for the hydranths. On the shoot, the hydrothecae are arranged in two practically opposite longitudinal rows, and each shoot internode has a pair of hydrothecae (Figure 3C) (Kosevich, 2008). The stolons are tube-shaped between the adjacent shoot bases and form a characteristic widening at the shoot base (Figures 3A, B). The perisarc covering the stolons and shoots is rigid and becomes thicker with the age of the colony part.

**Figure 6:**
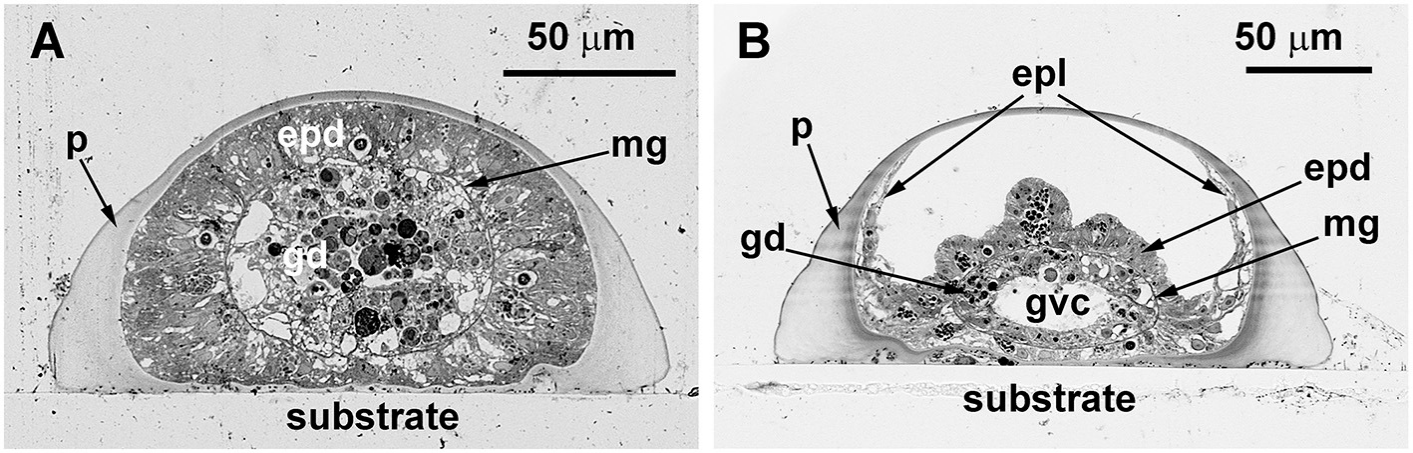
Cross-sections of the *Dynamena pumila* stolon in different parts. Semi-thin histological sections. **(A)** A cross-section of the stolon growing tip–soft tissues occupy the entire space within the perisarc tube and have permanent contact with its walls. **(B)** Cross-section of the stolon between the shoot bases—the coenosarc tube lies on the “bottom” of the perisarc tube and partly spreads on its side walls. epd, epidermis; epl, epidermal layer; gd, gastrodermis; gvc, gastro-vascular cavity; mg, mesoglea; and p, perisarc.

The stolons appear tube-like, but on each cross-section, they have a wide flat side that contacts the substrate and hemispheric surface opposing the substrate. The inner lumen within the stolon perisarc is practically oval. Therefore, the thickness of the perisarc differs along its circumference (Figure 6). The coenosarc of the stolons occupies the inner space of the perisarc tube completely, having permanent contact with the perisarc only in the distal growing parts of the stolon, i.e., in the growing tips (Figure 6A). In other parts of the stolon, the coenosarc tube has a smaller diameter; it lies on the “bottom” of the perisarc cavity and can contact the perisarc only during temporary widening. However, the epidermis of the coenosarc spreads over the sides of the perisarc tube in a one-cell layer (Figure 6B). Due to this, in the case of longitudinal disruption of the perisarc tube for ICC labeling, the epidermis of the stolons is inevitably traumatized, causing non-specific labeling.

**Figure 7:**
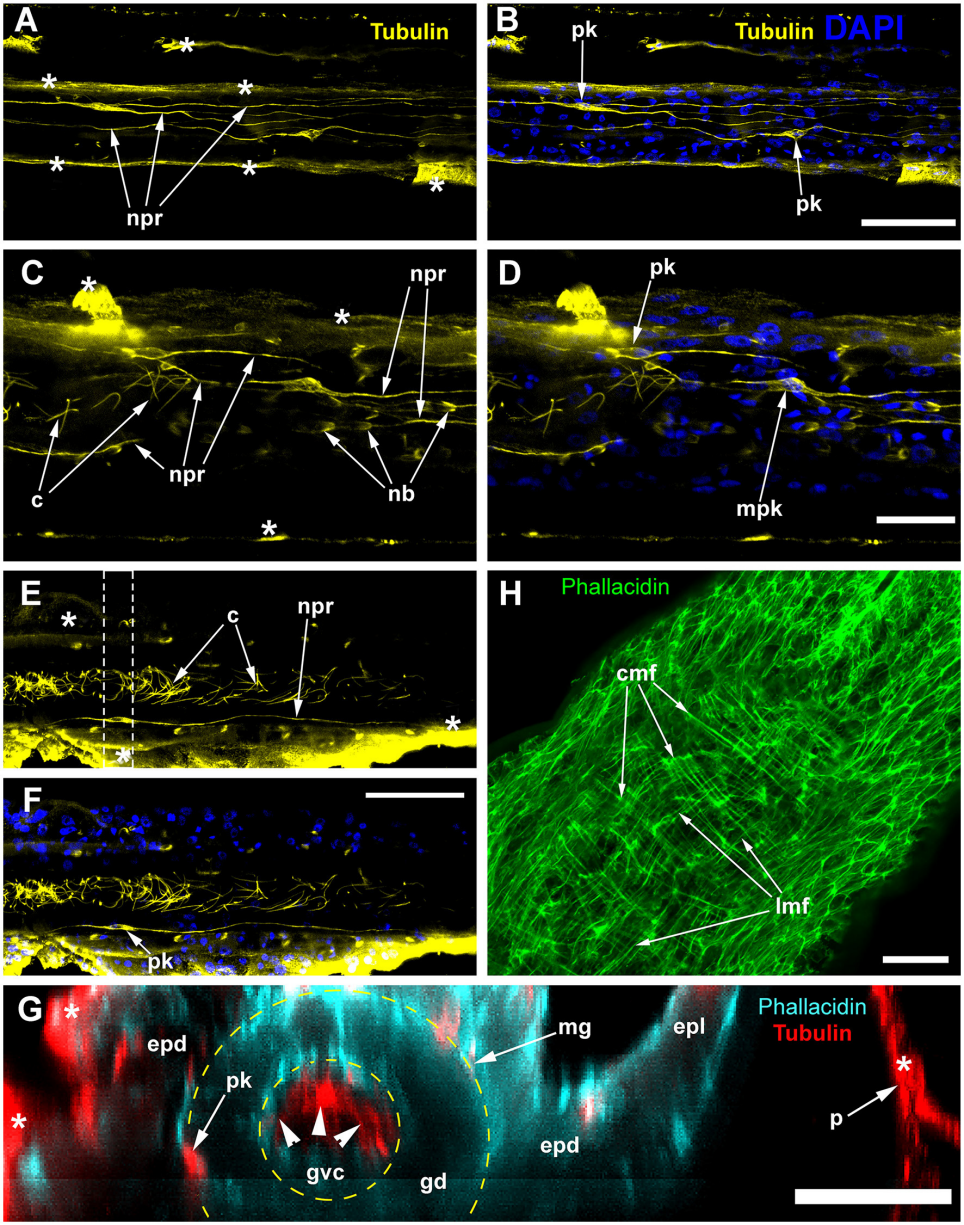
Alpha-tubulin-like immunoreactivity in the stolon of *Dynamena pumila*. Confocal laser scanning microscopy. Z-projections **(A–H)** after immunostaining against alpha-tubulin shown in yellow **(A–F)** or red **(G)**; nuclear staining (DAPI) shown in blue **(B, D, F)**; phallacidin staining shown in cyan **(G)** or green **(H)**. **(A–F)** Immunostaining against the alpha-tubulin of different parts of the stolon between the shoot bases: the nuclear staining confirms the visualization of the perikaria. **(G)** YZ projection (a virtual cross-section of the stolon) of the region in E between dashed lines, including the perikarion of the nerve-like cell [pk in **(F)**] located at the base of the epidermis over the mesoglea (compare with Figure 6B); yellow dashed circles mark the levels of the mesoglea and the border between the gastrodermis and gastro-vascular cavity. **(H)** Staining of the stolon growing tip with phallacidin showing the network of the muscle processes. c, cilia of the gastrodermal cells; cmf, circular muscular microfilaments; epd, epidermis; epl, epidermal layer; gd, gastrodermis; gvc, gastro-vascular cavity; lmf, longitudinal muscular microfilaments; mpk, perikaryon of the multipolar labeled cell; nb, nematoblast; npr, neurite-like process; and pk, perikarya of the labeled cells; white asterisks mark the artifacts, non-specific labeling of the traumatized tissue and disrupted perisarc; white arrowheads point to the cilia of the gastrodermal cells. **(A, B)** Maximum Z-projections of 24 optical sections with 0.15 μm step (overall section thickness −3.45 μm). **(C, D)** Maximum Z-projections of 36 optical sections with 0.1 μm step (overall section thickness −3.5 μm). **(E, F)** Maximum Z-projections of 73 optical sections with 0.2 μm step (overall section thickness −14.4 μm). H, maximum Z-projections of 38 optical sections with 0.2 μm step (overall section thickness −7,4 μm). In **(E, F)**, the optical sections pass parallel to the substrate practically through the middle of the stolon perpendicular to its side wall. Scale bars: **(A, B, E, F)**, 50 μm; **(C, D, G, H)**, 20 μm.

ICC labeling of the stolons with anti-α-tubulin antibodies visualized a system of cells with delicate processes, approximately 35–60 μm long (Figures 7A–F). Most of the processes run along the axis of the stolon, at a distance of ~6–8 μm from each other and form a loose network. The cell processes can contact one another or the soma of other labeled cells. Most of the alpha-tubulin-like immunoreactive cells look like bipolar neurons (Figures 7A, B, E–G). Multipolar neuron-like cells are rare and bear 1–2 long and 1–2 short processes (Figures 7C, D). No signs of cell condensation are revealed by the anti-alpha-tubulin labeling. The anti-α-tubulin antibodies visualized numerous nematoblasts within the stolon, although they were less numerous than in *C. multicornis*. Nematoblasts can also be seen in connection with the neurite-like processes of the alpha-tubulin-like immunoreactive cells (Figures 7A, C).

Labeling with phallacidin shows a system of longitudinal and circular muscular fibers. They can be distinguished most clearly within the growing tip and in the coenosarc just proximal to it (Figure 7H). These are the parts of the stolon that permanently undergo active contraction and pulsation (Wyttenbach, 1968; Beloussov, 1973, 1991; Kosevich, 2006).

**Figure 8:**
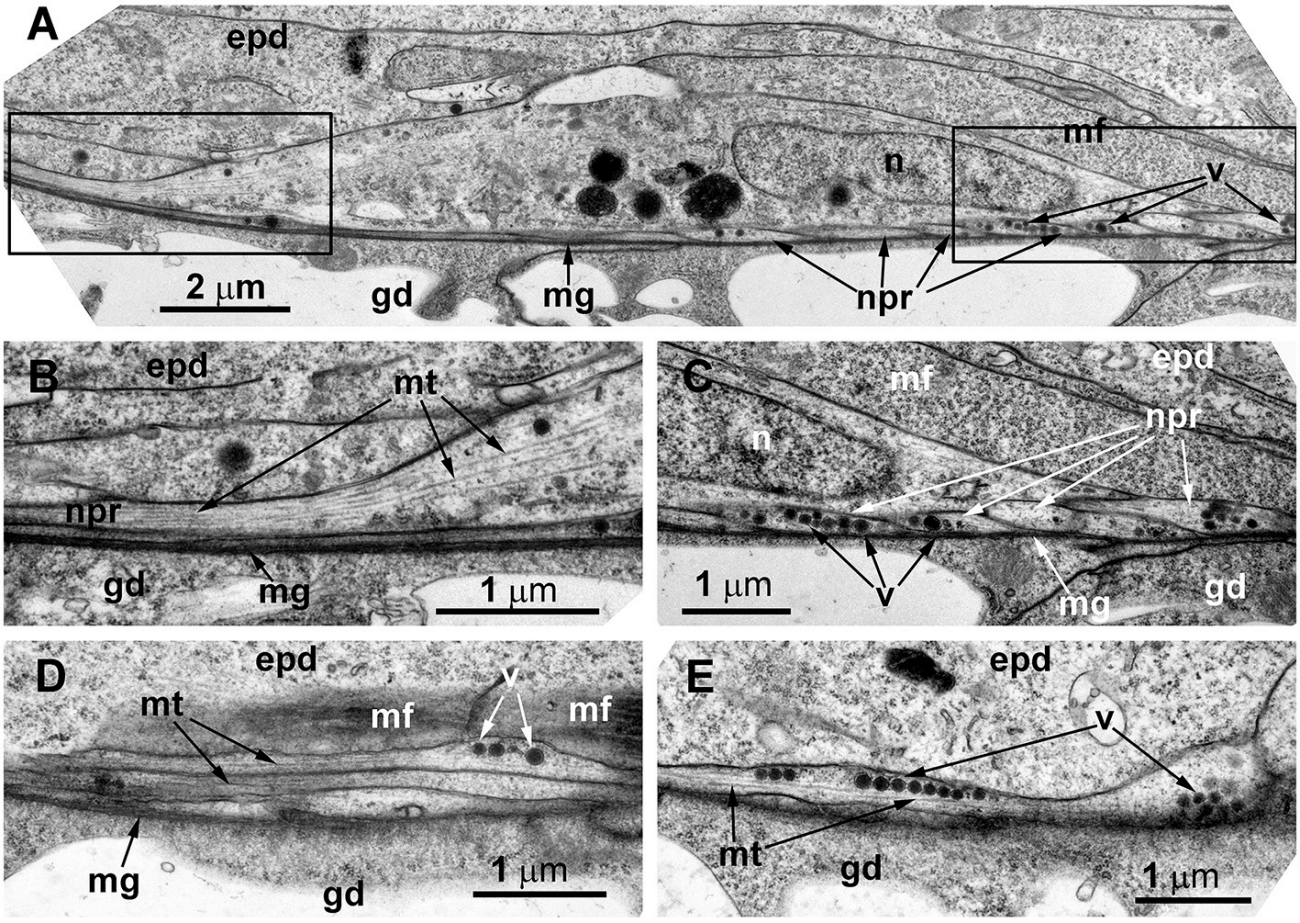
The basal part of the stolon epidermis of *Dynamena pumila* illustrating some ultrastructural features of the ganglion nerve cells. Longitudinal ultrathin section, TEM. **(A)** Soma of the neuron located below the muscular processes of epidermal cells and lying over the neurites, close to the mesoglea. **(B, C)** Enlarged view of the regions marked in A on the left **(B)** and right **(C)** sides. **(D, E)** Bundle of neurites with numerous microtubules and dense-core vesicles. epd, epidermis; gd, gastrodermis; mf, contractile myofibrils; mg, mesoglea; mt, microtubules; n, nucleus; npr, nerve process; and v, vesicles.

Ultrastructural study of the stolon in *D. pumila* confirmed the presence of nerve cells in the epidermis. The neurons are rare, and sometimes their structure was difficult to determine in the cross-sections. However, analysis of longitudinal sections clearly showed the presence of the nervous system in the stolons (Figure 8). The neurons are positioned in direct contact with the mesoglea, and contractile muscular processes of epidermal cells lie over the neurons. The somas of the neurons are elongated and contain few clear and dense-core vesicles, as well as numerous microtubules at the base of the processes (Figure 8A). The nuclei are flattened. Separate neurites or bundles of neurites run along the mesoglea and are characterized by numerous microtubules (Figures 8B–E) and dense-core vesicles 120–160 nm in diameter, often forming groups along the processes (Figures 8D, E).

### Obelia longissima

To confirm the presence of the nervous system elements in the coenosarc of the shoots, the internodes of the “giant” shoots (Kosevich, 1991b) underwent ICC labeling. This species is characterized by heavily branching shoots with a sympodial mode of growth. Like in *D. pumila*, rigid perisarc covers all parts of the colony. During the ontogeny of the colony in favorable conditions, the size of the newly forming shoot internodes gradually increases and can reach 2–5 mm in length and ~1 mm in diameter (Figures 3D, E). Therefore, it is easier to disrupt the perisarc of the internode with less damage caused to the coenosarc.

**Figure 9:**
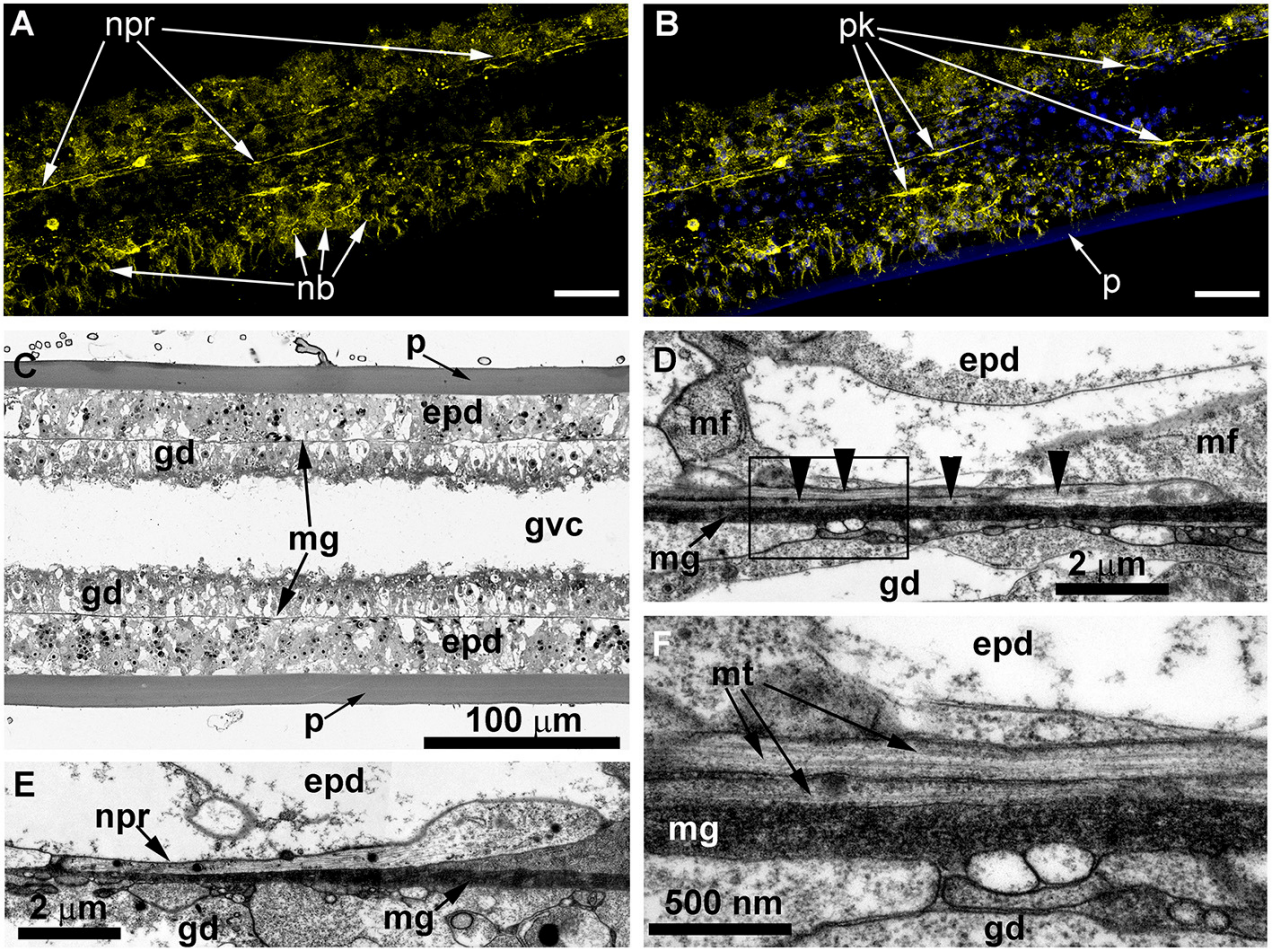
Details of the neuron-like cells in the coenosarc of *Obelia longissima* shoot internode, longitudinal optical **(A–C)**, and ultrathin **(D–F)** sections (TEM). **(A, B)** —z-projections after immunostaining against alpha-tubulin [shown in yellow **(A, B)**] and nuclear staining [shown in blue **(B**, superimposition with image **A)**] (confocal laser scanning microscopy); z-projections of 50 optical sections with 0.175 μm step (overall section thickness −8.575 μm). **(C)** Semi-thin histological section of the part of the internode. **(D–F)** The ultrastructure of the basal part of the epidermis showing the features of the neurites. epd, epidermis; gd, gastrodermis; mf, contractile myofibrils; mg, mesoglea; mt, microtubules; nb, nematoblast; and npr, nerve process; p, perisarc; pk, perikaryon of the labeled cell; black arrowheads point to the microtubules. Scale bar: a, B−50 μm.

ICC labeling with anti-α-tubulin antibodies revealed the presence of multiple alpha-tubulin-like immunoreactive cells (Figures 9A, B). In comparison with the other studied species, it appears that the number of immunoreactive cells in the coenosarc of *O. longissima* is greater, but the cell processes look shorter—~40–50 μm. The neurite-like processes appear highly delicate and do not form a clear network, as most of them run longitudinally along the internode axis at a distance of ~10 μm from each other.

**Figure 10:**
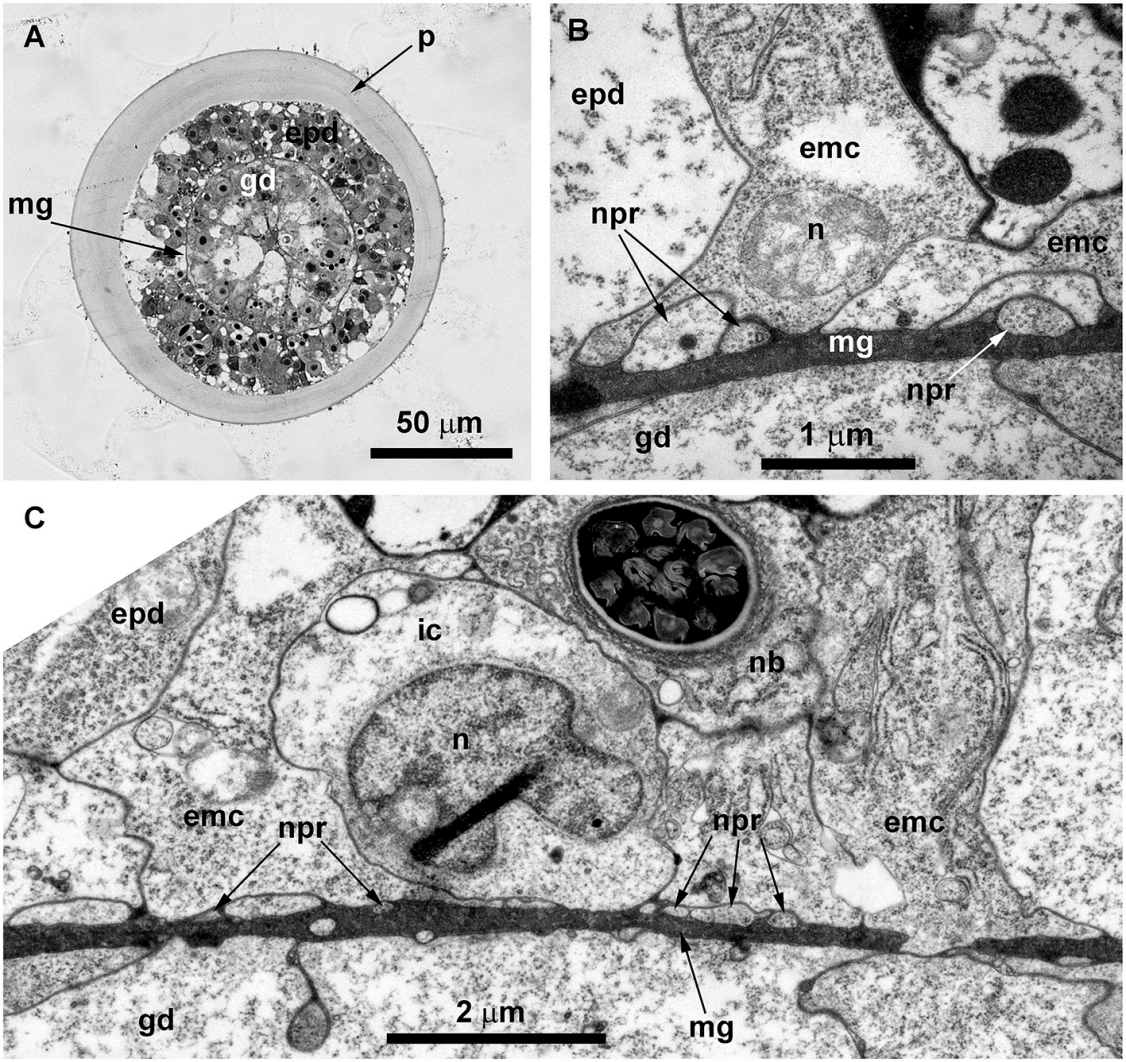
Histological **(A)** and ultrastructural **(B, C)** cross-sections of the shoot internode of *Obelia longissima*. **(A)** The cross-section of the entire internode; the situation when the specimen was fixed at the time-point when the gastro-vascular cavity was closed due to the periodic peristaltic wave of coenosarc contraction. **(B)** Neurites located over the mesoglea at the base of the epithelia-muscular epidermal cells. **(C)** Part of the cross-section of the epidermis base showing the spatial relationship between the neurites and the other cells of the epidermis. emc, epithelia-muscular cell; epd, epidermis; gd, gastrodermis; ic, i-cell; mf, contractile myofibrils; mg, mesoglea; mt, microtubules; nb, nematoblast (with nematocyst); npr, nerve process; and p, perisarc.

**Figure 11:**
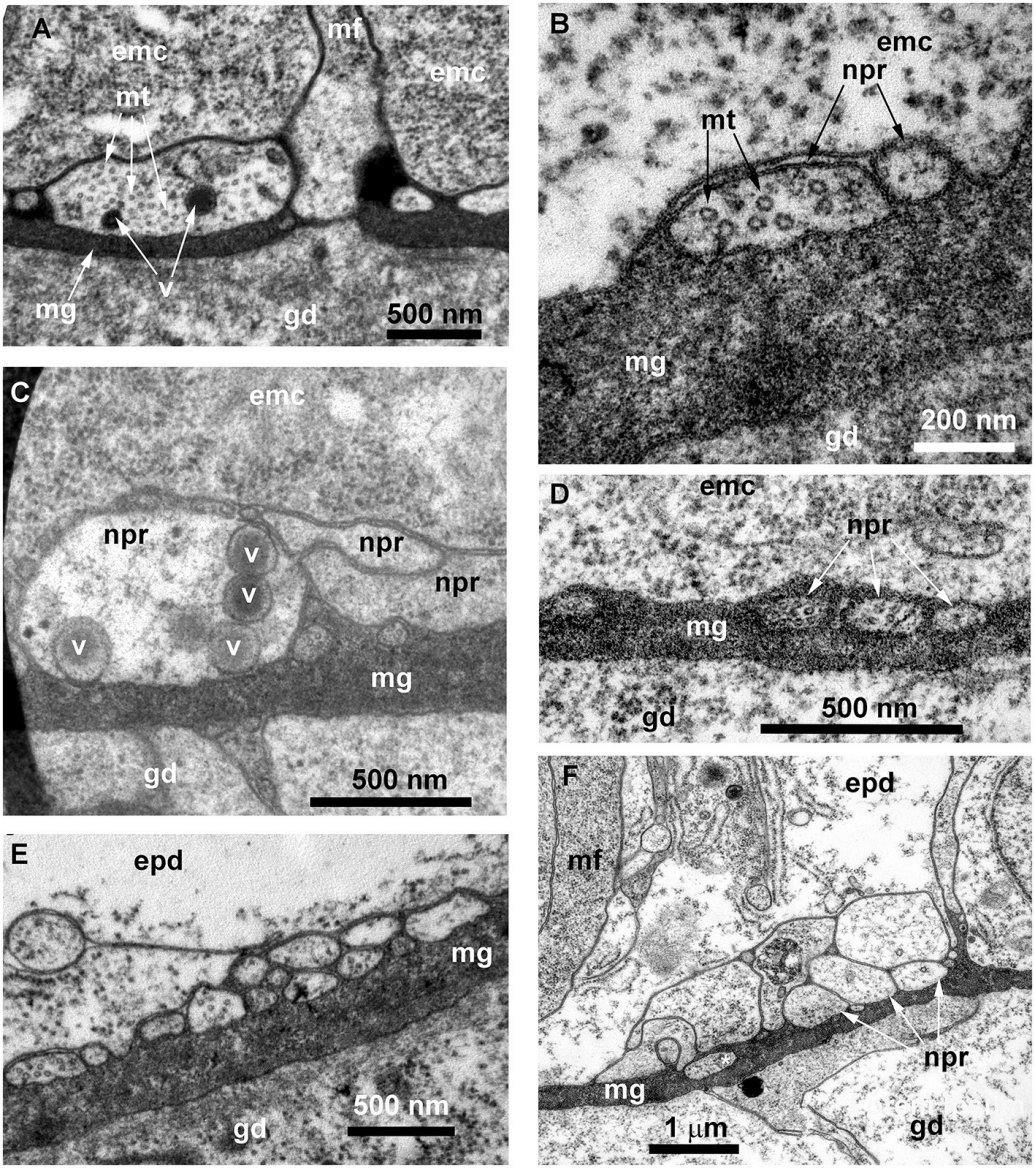
Details of the ultrastructure of the neurites visible at the cross-sections of the *Obelia longissima* shoot internode. Ultrathin sections, TEM. **(A–C)** A separate neurite **(A)** and a small bunch of neurites **(B, C)** showing the characteristic features of the nerve processes: the microtubules and the dense-core vesicles. **(D)** A small bunch of neurites submerged in mesoglea. **(E, F)** Flattened bundles of neurites between the mesoglea and the base of the epidermal cells. White asterisk marks the probable neurite submerged in mesoglea. emc, epithelia-muscular cell; epd, epidermis; gd, gastrodermis; mf, contractile myofibrils; mg, mesoglea; mt, microtubules; npr, nerve process; and v, vesicles.

Ultrastructural study of longitudinal and transverse sections of the shoot internode of *O. longissima* revealed ganglion nerve cells with characteristic features. Despite the greater outer diameter of the perisarc and the coenosarc of the internodes in “giant” shoots, the thickness of the two-layer wall of the coenosarc tube is comparable to other species belonging to the family Campanulariidae (Figures 9C, 10A). Therefore, the size of the cells is more or less constant. The ganglion nerve cells and their processes run over the mesoglea (Figures 9D–F) beneath the contractile processes of epitheliomuscular cells (Figures 9D–F, 10B, C). The neurites are characterized by the presence of numerous microtubules and dense-core vesicles (Figures 9D, E, 10B, 11). The latter look less numerous in comparison with the species described above. The neurites do not form pronounced bundles and are mostly found in groups of 2–3 processes (Figures 9D, F, 10B, C, 11B, C). When more processes are found together, they form a bundle flattened along the mesoglea (Figures 10C, 11E, F). Quite often, the neurites appear to be submerged in the mesoglea (Figures 10C, 11D, F).

## Discussion

This is one of the first studies to visualize the presence of a colonial nervous system in the coenosarc of colonial representatives belonging to the orders Anthoathecata and Leptothecata, the two most numerous taxa of Hydrozoa. The results of immunocytochemical labeling against alpha-tubulin and RF-amide coincide almost completely and confirm that the labeled cells are nerve cells. Ultrastructural investigation of the stolon and shoot coenosarc in all tree species supports the presence of nerve elements in the epidermis alone. The observed cells have the main characteristic features proposed to be the key ultrastructural features for identifying a cell as a nerve cell (Jha and Mackie, 1967; Stokes, 1974a): both the cytoplasm of the cell body and the long cell processes contain numerous microtubules, as along with dense-core and clear vesicles of characteristic sizes. In some cases, a short basal part of the cilia was found (not shown) within the somas of such cells.

In hydrozoans, three main cell types belong to the nervous system: ganglion cells, sensory cells, and nematocytes, which are sometimes referred to as mechanosensory cells (Watanabe et al., 2009). Ganglion cell perikaria are located at the base of the epidermis and never reach the surface of the epithelium, and ganglion cells have two or more processes. In some cases, ganglion cells were shown to possess “rudimentary” cilia (Westfall, 1973a). The soma of the sensory cell spans the entire depth of the epidermis and has direct contact with the outer environment (Westfall and Kinnamon, 1978; Koizumi et al., 1988; Havrilak et al., 2017). Moreover, sensory cells bear cilia on the apical surface. All these data allow the nerve cells in the coenosarc of a colony, which were described in the present study, to be recognized as ganglion nerve cells known for most of the hydrozoan species. As the coenosarc of colonial hydroids is permanently covered by rigid perisarc, there are no sensory cells in this part of the hydroid body.

The fact that no nerve elements were found in the coenosarc of sessile colonial hydroids for a long time can be attributed to two reasons. First, the perisarc that covers the “colonial” tissues hinders the application of ICC staining with antibodies. That is why, for example, investigation of the hydroid planula-larva settlement and metamorphosis showed the development of a nervous system within the primary hydranth of *Hydractinia echinata* and the absence of nerve elements in the stolons (Plickert et al., 1988). The same situation was encountered when *Pennaria tiarella* settlement and metamorphosis were studied: nerve elements were not detected through ICC staining with anti-RF-amide antibodies in the primary polyp stack (Martin, 2000). In both examples, the absence of staining can be explained by the development of the perisarc over the stolon and stack tissue.

Second, compared with the tissues of the zooids, the ultrastructure of the coenosarc of the stolons and shoots is less studied. The minute dimensions of the neurons and their neurites within the coenosarc, combined with their loose spatial distribution at the base of the epidermis along the mesoglea, can cause these structures to be overlooked (see Josephson, 1961b; Spencer, 1974). In most of the hydrozoans studied (and in cnidarians on the whole), the neurons and their neurites within the epidermis of zooids were located above the sheet of the contractile filaments of the epitheliomuscular cells, forming a dense network of prominent nerve bundles (Westfall, 1973a,b; Thomas and Edwards, 1991; Golz, 1994; Cole et al., 2020). These neurites contribute to the coordination of zooid behavior. The location of the neurons and their neurites below the contractile filaments of the coenosarc epitheliomuscular cells does not preclude the possibility that the colonial nervous system coordinates muscular contraction. It is possible that translocation of the nerve elements underneath the muscular processes is correlated with the active migration of diverse cells along the base of the epidermal layer: nematoblasts, gland cells, precursors of germ cells, etc. (Aizenshtadt and Polteva, 1981; Kosevich, 2013). In such a case, the nerve elements will not be disturbed. However, ICC investigations revealed a situation when nematoblasts differentiating and migrating within the coenosarc appeared to be positioned along the neurites. A possible explanation is that neurites are somehow involved in directing the migration of nematoblasts and other cells.

The feeble development of the colonial nervous system in sessile colonial hydrozoans can be attributed to the absence of intracolonial coordination of behavior. Colonial (modular) invertebrates are decentralized organisms (Marfenin, 2016); they have no central nervous system, and they have no center for coordinating the behavior of the entire organism. That is why no rapid colonial response to local stimuli was observed in several studies (e.g., Josephson, 1961a,b; Mackie, 1965; Morin and Cooke, 1971; Mayorova and Kosevich, 2013). A colonial nervous system was described for representatives of Siphonophorae, the motile modular hydrozoans (e.g., Mackie, 1965; Jha and Mackie, 1967; Grimmelikhuijzen et al., 1986), for whom coordinated behavior is essential. Furthermore, there is a view that nerves are not always necessary for coordinated behavior (Mackie, 1990), and conduction can be carried out by the epithelial layer cells or the muscular bases of epithelial cells (Josephson, 1961b, 1965; Mackie, 1965; Spencer, 1974) (epithelial conduction). This conduction spreads at a somewhat slower pace, and for now, nothing is known about the functions that epithelial conduction can perform.

The primary data about the described colonial nervous system without any functional studies give no clear idea about the functions of this system in hydroids. This system utilizes an RF-amide neuropeptide, which is commonly found in cnidarians (Grimmelikhuijzen et al., 1991). RF-amide neuropeptides belong to a group of FMRF-amides discovered in mollusks (Price and Greenberg, 1977). FMRF-amide was shown to have an excitatory effect on cnidarian muscles (Kass-Simon and Pierobon, 2007). Hence, it is involved in the activity of zooids. However, the coenosarc displays limited activity: periodical peristaltic waves pass along the stolon and shoot coenosarc (Marfenin and Dementyev, 2019) and the growing tips of the stolons and shoots demonstrate growth pulsations (Kosevich, 2006). As the colonial body constitutes a branching tube, the coenosarc cannot contract (shorten its length), so the longitudinally oriented epidermal muscle processes appear functionless. The gastrodermal muscle processes oriented along the circumference of the coenosarc cause the tube diameter to contract. The epidermal and gastrodermal contractile processes of the hydrozoan polypoid stage belong to the smooth muscle type and can contract spontaneously as a response to stretching and not just in response to stimuli from the nervous system (Webb, 2003). This means that the peristaltic waves of relaxation and contraction of the coenosarc may not require nerve control: they can be activated by fluid flow from the zooids (Marfenin and Dementyev, 2018) or the pulsating zones behind the growing tips (Hale, 1960). The flow from the contracted zooid causes the neighboring coenosarc to widen and the circular muscle fibers to stretch. Subsequently, the circular muscle fibers will respond in a while by shortening. Therefore, it is possible that the colonial nervous system described is not indispensable for the functioning of the food transport system, which is the main integrating mechanism of a hydroid colony (Marfenin, 2015).

The neurites of the colonial nervous system were found in the growing tips of stolons. Therefore, it is possible that the described colonial nervous system may be involved in regulating the functioning of the tip: for example, in the regulation of growth pulsations (Kosevich, 2006). Growth pulsations are based on two consecutive processes. The first step of pulsation is caused by a reorientation of the epidermal sheet cells due to an increase in turgor within the cells (Beloussov, 1973; Zaraisky et al., 1984). The second step involves a decrease in the gastrodermal tube diameter, which causes its elongation (Kosevich, 2006). The second step obviously relies on muscular contraction. Therefore, we can hypothesize that at least the second step of the growth pulsations is under the control of the nervous system. Moreover, the growth pulsations possess a regular rhythm, and it cannot be denied that the colonial nervous system may play the role of a pacemaker.

The organization of a hydroid colony is based on a repetitive morphogenesis of colony elements during the entire ontogeny of the organism. Hydroids, like all cnidarians, possess an outstanding ability for regeneration, and the growth of a colony strongly resembles a continuous regeneration process (Kosevich, 1991a, 1996). In bilaterians, the peripheral nervous system plays diverse roles in the regulation of embryonic development, homeostasis of tissues, and nerve-dependent regeneration (Kaucká and Adameyko, 2014). Another possible role of the colonial nervous system in hydroids not connected with the coordination of behavior is participation in the morphogenetic program of the growing tips. The available data do not contradict such a possibility.

## Data availability statement

The raw data supporting the conclusions of this article will be made available by the authors, without undue reservation.

## Ethics statement

Ethical review and approval was not required for the study of animals in accordance with the local legislation and institutional requirements.

## Author contributions

IK: conceptualization, methodology, investigation, resources, writing—original draft preparation, review and editing, and visualization.
